# Dutch healthcare reform: did it result in performance improvement of health plans? A comparison of consumer experiences over time

**DOI:** 10.1186/1472-6963-9-167

**Published:** 2009-09-17

**Authors:** Michelle Hendriks, Peter Spreeuwenberg, Jany Rademakers, Diana MJ Delnoij

**Affiliations:** 1 NIVEL (Netherlands Institute for Health Services Research), Utrecht, the Netherlands; 2 Centre for Consumer Experience in Healthcare, Utrecht, the Netherlands; 3 TRANZO (Scientific Centre for Transformation in Care and Welfare), Faculty of Social and Behavioural Sciences, Tilburg University, Tilburg, the Netherlands

## Abstract

**Background:**

Many countries have introduced elements of managed competition in their healthcare system with the aim to accomplish more efficient and demand-driven health care. Simultaneously, generating and reporting of comparative healthcare information has become an important quality-improvement instrument. We examined whether the introduction of managed competition in the Dutch healthcare system along with public reporting of quality information was associated with performance improvement in health plans.

**Methods:**

Experiences of consumers with their health plan were measured in four consecutive years (2005-2008) using the CQI^® ^health plan instrument 'Experiences with Healthcare and Health Insurer'. Data were available of 13,819 respondents (response = 45%) of 30 health plans in 2005, of 8,266 respondents (response = 39%) of 32 health plans in 2006, of 8,088 respondents (response = 34%) of 32 health plans in 2007, and of 7,183 respondents (response = 31%) of 32 health plans in 2008. We performed multilevel regression analyses with three levels: respondent, health plan and year of measurement. Per year and per quality aspect, we estimated health plan means while adjusting for consumers' age, education and self-reported health status. We tested for linear and quadratic time effects using chi-squares.

**Results:**

The overall performance of health plans increased significantly from 2005 to 2008 on four quality aspects. For three other aspects, we found that the overall performance first declined and then increased from 2006 to 2008, but the performance in 2008 was not better than in 2005. The overall performance of health plans did not improve more often for quality aspects that were identified as important areas of improvement in the first year of measurement. On six out of seven aspects, the performance of health plans that scored below average in 2005 increased more than the performance of health plans that scored average and/or above average in that year.

**Conclusion:**

We found mixed results concerning the effects of managed competition on the performance of health plans. To determine whether managed competition in the healthcare system leads to quality improvement in health plans, it is important to examine whether and for what reasons health plans initiate improvement efforts.

## Background

Nowadays, several western countries have introduced some form of managed competition in their healthcare system with the aim to accomplish a more efficient and more demand-driven healthcare [1,2]. For these overall aims to be achieved, the performance of both healthcare providers and health plans has to be assessed and publicly reported [3]. Consumers/patients need this quality information in order to be able to act as informed and critical decision-makers on both the healthcare market (choosing between healthcare providers) and the health insurance market (choosing between health plans). Health plans are expected to use the quality information on healthcare providers to differentiate between healthcare providers when contracting health care selectively on the health purchaser market. Both health plans and healthcare providers should use the information on their own performance to monitor the quality of their service and/or care and to initiate quality-improvement projects. Generating and reporting of comparative healthcare information has therefore become an important quality-improvement instrument in several countries [4-6].

Fung et al. has shown that public reporting of quality information does indeed stimulate hospitals to initiate quality-improvement projects and that some consumers use this information when choosing a hospital [3]. Hibbard et al. showed that publicly reporting quality information stimulated hospitals to start quality-improvement projects but only for those areas where the quality of health care was inferior [7,8]. Two years later, the quality of hospital care had improved for these areas and this improvement was more profound for hospitals that performed worse than expected at baseline.

The studies of Fung and Hibbard et al. focused on hospital care. In the present study, we will focus on the question whether the introduction of managed competition within the healthcare system along with reporting of quality information is associated with quality improvement in health plans. Concerning health plans, researchers so far have only investigated whether consumers use quality information when choosing a health plan. It appeared that consumers do use the information and tend to choose better performing health plans [3,9]. Empirical studies on the effects of the introduction of managed competition and the publication of quality information on the performance of health plans is, however, lacking.

To answer this question, we used data on the performance of health plans in the Netherlands. In January 2006, the Dutch government introduced more managed competition in their healthcare system by enacting a new health insurance law. The most important changes in and characteristics of the Dutch health insurance system are presented in Table 1. A basic obligatory insurance, covering the entire population, was introduced. All consumers have a free choice between insurance companies during annual open enrolment periods (November-January). Health plans are obliged to accept every citizen for the basic package and are no longer allowed to select favourable risks or to differentiate the premium and conditions according to (proxies for) risk [10]. Health plans can negotiate with the healthcare providers on the price, content and organisation of the care and do not have to enter into a contract with every provider. In addition, they can offer collective arrangements to their insured against a reduced nominal premium. These reforms should make switching health plans easier and are supposed to lead to a more demand-driven health care system that is cheaper and of higher quality [11].

**Table 1:** The Dutch health insurance system after the insurance reform of 1 January 2006

**Health insurance law**	Introduced on 1 January 2006
	Abolition of distinction between private and public insurance
	Insurance under private law with public limiting conditions
	Obligation for every citizen to take out health insurance in form of basic package
	Risk adjustment
	
**Insurance policy**	Free choice between health plans during annual open enrolment periods
	Basic insurance package which is identical for everybody and health plans are obliged to accept everybody against the same premium and conditions
	Choice between in-kind and restitution policy
	Health plans have the possibility to contract health care selectively
	Possibility for citizens to take out an complementary insurance. Health plans are not obliged to accept everybody
	Choice of deductible (minimum €100, max. €500); from 2008 obligatory deductible of at least €155
	No-claim reimbursement of up to 255 Euros; abolished in 2008
	Collectives (via work or other) get premium reduction up to 10%

In addition, the performance of health plans is assessed annually in the Netherlands using the standardized CQI^® ^health plan instrument 'Experiences with Healthcare and Health Insurer'. Consumer Quality Index (CQ-index or CQI) instruments assess the quality of health care seen from the consumer's perspective, and measure consumers' experiences instead of inquiring after their satisfaction [12,13]. The experiences of consumers/patients with their health plan have been measured in four consecutive years (2005 to 2008) starting the year before the introduction of the new insurance law [14-17]. In all years, the resulting comparative quality information was published on the healthcare portal  (Make better choices), which is an initiative of the Dutch Ministry of Health, Welfare and Sport. In addition, a press release was published mentioning the quality aspects that in general need improvement the most. The health plans received a confidential company report in which their performance was compared with the average across all health plans.

We examined whether the performance of the health plans improved with the introduction of managed competition in the Dutch health insurance system and the coinciding publication of comparative quality information by looking at the changes over the years in consumer experiences with 2005 as baseline measurement. In line with the studies of Hibbard et al [7,8,18], we hypothesized that all health plans would improve their performance but that the changes over the years would be more profound for identified areas of improvement and for health plans with an inferior performance at the first measurement.

## Methods

### Available data

Experiences of consumers with the provided health care and the services of their health plan were measured in four consecutive years (2005-2008) using the standardized CQI^® ^instrument 'Experiences with Healthcare and Health Insurer'. Each year, this questionnaire was sent to a different random sample of insured. The CQI health-plan instrument consists of items on health-plan services and received healthcare in the past twelve months. It contains 54 core items on consumer experiences, four global ratings (family physician, specialist, healthcare, and health plan), one item on the likelihood to recommend the health plan to friends and family, and several items on consumer characteristics. The questionnaire is partly a transformation of the CAHPS 3.0 Adult Commercial Questionnaire [19]. For this study, we focused on seven quality aspects of the health-plan services (See Table 2): the global rating of health plan, conduct of employees, health plan information, access to call centre, getting the needed help from the call centre, reimbursement of claims and transparency of (co)payment requirements.

**Table 2:** Quality aspects of health-plan services

**Quality aspect**	**Number of items**	**Scale**
General rating of health plan	1	0-10
Conduct of employees	5	1-4
Health plan information	3	1-3
Access to call centre	1	1-3
Getting the needed help from call centre	1	1-4
Reimbursement of claims	2	1-4
Transparency of (co)payment requirements	1	1-4

Note. Scales were obtained from exploratory factor analysis of the experience items; mean scores were calculated.

### Published results

In each year the following statistical analyses were performed [14-17]. The respondents and non-respondents were compared concerning age and sex in order to determine whether a response bias occurred. Using multilevel linear regression analyses (consumers' experiences were nested within health plans), means with comparison intervals (1.39 × standard error; [20]) were calculated per aspect and per health plan while adjusting for consumers' age, education and self-reported health status. Next, health plans were divided in three groups by determining whether the comparison interval overlapped with the overall mean of all health plans. This classification of health plans was published on the website  using stars: * = below average (comparison interval lies below overall mean), ** = average (comparison interval overlaps with overall mean) and *** = above average (comparison interval lies above overall mean).

In addition, the research institute (NIVEL) published a press release each year on their own website highlighting the most important results of the study. The press release mentioned among other things on which quality aspects the overall performance of the health plans was inferior. We expected that health plans would especially try to improve these aspects. The following areas of improvement were identified in 2005: transparency of (co)payment requirements, access to call centre and health plan information. The results of 2005 were published in at least five (national) news papers and the media also covered the launch of the information on the website .

### Secondary statistical analyses

We combined the data of the four years and performed another set of multilevel regression analyses with three levels: respondent, health plan and year of measurement. Per year and per quality aspect, we estimated the overall mean across health plans while adjusting for the consumers' age, education and self-reported health status. To determine whether the performance of the health plans improved over the years, we tested for linear and quadratic time effects and compared the performance in 2005 with the performance in 2008 using chi-squares.

In order to determine whether the changes over the years were more profound for quality aspects that were identified as aspects that needed improvement most and for health plans with an inferior performance at the first measurement, we focused on the differences between 2005 and 2008. We choose the longest time frame possible, because health plans need time to implement and put into effect improvement efforts. First, we examined whether the performance of health plans increased more often on quality aspects that were mentioned as important areas of improvement in the 2005-press release than on the other quality aspects. Then, we investigated whether the differences in performance between 2005 and 2008 depend upon the performance of health plans in 2005 as published on the Dutch website . Health plans who did not participate in the 2005-study are therefore excluded from these analyses. For each group of health plans (below-average scoring, average scoring and above-average scoring health plans), we estimated the mean per aspect for 2005 and 2008 and tested whether the differences were statistically significant using chi-squares.

## Results

### Respondents

In 2005, 13,819 respondents (response = 45%) of 30 health plans filled out the questionnaire. The number of respondents per health plan varied from 167 to 1,287 (mean = 461; SD = 223.80). In 2006, 8,266 respondents (response = 39%) of 32 health plans filled out the questionnaire, and the number of respondents per health plan varied from 205 to 348 (mean = 258; SD = 38.58). In 2007, the questionnaire was filled out by 8,088 respondents (response = 34%) of 32 health plans, and the number of respondents per health plan varied from 154 to 353 (mean = 253; SD = 43.11). In 2008, the questionnaire was filled out by 7,183 respondents (response = 31%) of 32 health plans, and the number of respondents per health plan varied from 170 to 313 (mean = 224; SD = 36.73). The large differences in number of respondents per health plan in 2005 were due to mergers between health plans during the course of the study.

Table 3 shows the mean age and percentage of male of the respondents and non-respondents. In all years, the respondents were older than the non-respondents. In 2005, 2006 and 2007, more women than men filled out the questionnaire.

**Table 3:** Comparison of person characteristics between respondents and non-respondents

	**Respondents**	**Non-respondents**	**Difference**
**2005**			
Age (mean; SD)	49.5 (16.5)	43.5 (16.2)	F = 1035.92***
Sex (% male)	52	61	χ^2 ^= 240.44***
			
**2006**			
Age (mean; SD)	50.7 (16.6)	44.0 (16.8)	F = 824.35***
Sex (% male)	47	55	χ^2 ^= 107.78***
			
**2007**			
Age (mean; SD)	51.8 (16.9)	45.0 (17.0)	F = 883.50***
Sex (% male)	44	53	χ^2 ^= 165.72***
			
**2008**			
Age (mean; SD)	49.1 (18.0)	47.0 (16.7)	F = 72.76***
Sex (% male)	51	50	χ^2 ^< 1, ns

Note. *** p < .001.

### Overall changes in performance

Table 4 shows that the performance of the total group of health plans changed significantly over the years on all quality aspects. The performance on conduct of employees and transparency of (co)payment requirements improved from 2005 to 2008 leading to a significant better performance in 2008 than in 2005 (both χ^2^'s > 8.90; p < .01). Concerning access to call centre, getting the needed help from the call centre and the reimbursement of claims, the performance of health plans first declined from 2005 to 2006 and than increased. For these three quality aspects, we found no significant differences in performance between 2005 and 2008 (all χ^2^'s < 1.45). The performance on health plan information increased from 2005 to 2006 and than stabilized and the health plan information was better in 2008 than in 2005 (χ^2 ^= 16.93; p < .001). The general rating of health plans increased from 2005 to 2007 and then decreased somewhat in 2008, but the general rating in 2008 was still significantly higher than in 2005 (χ^2 ^= 8.59; p < .01). It is important to note that the changes in performance over the years are in general small.

**Table 4:** Results of multilevel analyses: estimated mean and standard error per quality aspect and chi-squares indicating the change over years for all health plans

**Quality aspect**	**M**	**SE**	**Time effects**	
			
			**Linear**	**Quadratic**
**General rating of health plan**			10.68**	33.67***
2005	7.53	0.06		
2006	7.66	0.05		
2007	7.75	0.05		
2008	7.66	0.05		
				
**Conduct of employees**			19.62***	0.19
2005	3.50	0.02		
2006	3.52	0.02		
2007	3.57	0.02		
2008	3.58	0.02		
				
**Health plan information**			15.56***	12.37***
2005	2.63	0.02		
2006	2.70	0.01		
2007	2.72	0.02		
2008	2.71	0.02		
				
**Access to call centre**			10.59**	20.81***
2005	2.56	0.04		
2006	2.36	0.04		
2007	2.59	0.03		
2008	2.60	0.03		
				
**Getting the needed help from call centre**			0.98	5.04*
2005	3.40	0.03		
2006	3.28	0.03		
2007	3.41	0.03		
2008	3.38	0.04		
				
**Reimbursement of claims**			1.27	4.50*
2005	3.67	0.02		
2006	3.60	0.03		
2007	3.68	0.02		
2008	3.67	0.02		
				
**Transparency of (co)payment requirements**			7.35**	6.33*
2005	2.68	0.03		
2006	2.68	0.04		
2007	2.67	0.04		
2008	2.79	0.04		

Note. * p < .05; ** p < .01; *** p < .001.

### Comparison of overall changes in performance between quality aspects

Three areas of improvement were mentioned in the 2005-press release, i.e., transparency of (co)payment requirements, access to call centre and health plan information. Health plans performed significantly better in 2008 than in 2005 on two of these aspects, namely health plan information and transparency of (co)payment requirements. The performance on access to call centre did not differ between 2005 and 2008. Health plans performed significantly better in 2008 than in 2005 on two out of four aspects that were not identified as areas of improvement, namely general rating of health plan and conduct of employees. The overall performance of health plans on the two other aspects, getting the needed help from the call centre and the reimbursement of claims, did not differ between 2005 to 2008. In other words, the performance of health plans did not improve substantially more often for the quality aspects that were identified as important areas of improvement in the press release.

### Comparison of changes in performance between health plans

Table 5 shows the number of health plans that scored below average, average and above average on each of the quality aspects in 2005. In Table 6, the estimated mean score of each group of health plans (below-average scoring, average scoring, and above-average scoring health plans) is presented per quality aspect for 2005 and 2008. Also, the chi-squares of possible differences between 2005 and 2008 are given.

**Table 5:** Number of health plans who scored below average, average and above average in 2005

**Quality aspect**	**Below average**	**Average**	**Above average**
General rating of health plan	17	2	11
Conduct of employees	7	14	9
Health plan information	4	20	6
Access to call centre	8	10	12
Getting the needed help from call centre	5	17	8
Reimbursement of claims	9	10	11
Transparency of (co)payment requirements	6	18	6

**Table 6:** Results of multilevel analyses: estimated mean and standard error per quality aspect in 2005 and 2008 and chi-squares indicating the time effects for health plans who scored below average, average or above average in 2005

	**2005**		**2008**		**Time effect**
	**M**	**SE**	**M**	**SE**	
**General rating of health plan**					
below average score in 2005	7.30	0.04	7.52	0.04	17.60***
average score in 2005	7.53	0.11	7.51	0.15	0.02
above average score in 2005	7.90	0.05	7.88	0.05	0.11
					
**Conduct of employees**					
below average score in 2005	3.34	0.02	3.52	0.05	15.38***
average score in 2005	3.49	0.02	3.55	0.03	5.55*
above average score in 2005	3.65	0.02	3.67	0.03	0.64
					
**Health plan information**					
below average score in 2005	2.54	0.02	2.71	0.04	16.96***
average score in 2005	2.61	0.01	2.72	0.02	22.61***
above average score in 2005	2.75	0.02	2.75	0.03	0.05
					
**Access to call centre**					
below average score in 2005	2.26	0.04	2.40	0.04	4.26*
average score in 2005	2.53	0.03	2.58	0.04	0.70
above average score in 2005	2.75	0.03	2.72	0.03	0.29
					
**Getting the needed help from call centre**					
below average score in 2005	3.13	0.04	3.23	0.08	1.43
average score in 2005	3.37	0.02	3.34	0.04	0.49
above average score in 2005	3.60	0.03	3.54	0.06	1.03
					
**Reimbursement of claims**					
below average score in 2005	3.51	0.02	3.65	0.03	16.53***
average score in 2005	3.68	0.02	3.64	0.03	1.01
above average score in 2005	3.79	0.02	3.70	0.03	9.19**
					
**Transparency of (co)payment requirements**					
below average score in 2005	2.49	0.04	2.65	0.07	3.89*
average score in 2005	2.63	0.03	2.75	0.04	5.80*
above average score in 2005	2.95	0.04	3.05	0.06	1.81

Note. * p < .05; ** p < .01; *** p < .001.

It appeared that the performance on getting the needed help from the call centre did not differ significantly between 2005 and 2008 for health plans that scored either below average, average or above average in 2005. For conduct of employees, health plan information and transparency of (co)payment requirements, the performance of both below-average scoring and average scoring health plans increased from 2005 to 2008, while the performance of above-average scoring health plans did not change significantly. The performance of below-average scoring health plans also improved from 2005 to 2008 on access to call centre and general rating of health plan, while the performance of average and above-average scoring health plans did not change significantly. For reimbursement of claims the following picture emerged: health plans that scored below average in 2005 increased their performance from 2005 to 2008, the performance of average scoring health plans did not change significantly, whereas the performance of above-average scoring health plans declined from 2005 to 2008.

## Discussion

The aim of the present study was to determine whether the introduction of managed competition in the Dutch healthcare system and the coinciding publication of comparative quality information on health plans was associated with performance improvement in health plans. Experiences of consumers with their health plan were measured in four consecutive years (2005 to 2008) starting the year before the introduction of a new health insurance law [14-17]. In all years, the resulting comparative quality information was published on a Dutch website along with a press release mentioning the quality aspects that in general needed improvement the most. We expected that the performance of all health plans would improve over the years, endorsing the expected effects of managed competition. Moreover, following Hibbard et al., we hypothesized that the improvements over the years would be more profound for quality aspects that needed improvement most and for health plans that performed inferior at the first measurement (year 2005) [7,8,18].

When we look at the changes in performance between 2005 and 2008, the expected overall improvement in performance was found for only four out of seven quality aspects, namely general rating of health plans, conduct of employees, health plan information and transparency on (co)payment requirements. For three other aspects (i.e., access to call centre, getting the needed help from the call centre and the reimbursement of claims), we found that the overall performance first declined from 2005 and 2006 and then increased from 2006 to 2008; the performance of health plans was, however, not significantly better in 2008 than in 2005.

The decline in overall performance from 2005 to 2006 on these three quality aspects can be explained as followed. In January 2006, the Dutch government enacted the new insurance law. The new law brought about several changes for Dutch citizens and created turmoil within the Dutch population. In the beginning of 2006, much more consumers than usual telephoned their health plan for extra information decreasing the access of the call centre and making it more difficult for health-plan employees to provide the needed help. The administrative burden associated with the introduction of the new health insurance law could explain the decreased performance concerning reimbursement of claims. However, after the first year of the new health insurance system, the health plans did not appear to be able to improve the performance of the call centre and the reimbursement of claims to a level higher than in 2005.

Above-mentioned results also indicate that the overall performance of health plans did not improve more often for quality aspects that were identified as important areas of improvement in 2005 (i.e., transparency of (co)payment requirement, access to call centre and health plan information) than for the other quality aspects. In short, the introduction of managed competition in the Dutch healthcare system along with the publication of comparative quality information only had the assumed positive effects on the overall performance of health plans for a subset of quality aspects.

Next, we examined whether health plans that performed below average in 2005 improved their performance more often than health plans that did not perform below average in that year. On most (six out of seven) aspects the performance of below-average scoring health plans increased more than the performance of average and/or above-average scoring health plans. In other words, the idea that health plans who scored relatively low in 2005 would try harder to improve their performance than health plans who scored relatively high was confirmed [7,8]. It is, however, important to keep in mind that relatively bad-performing health plans had more possibilities for improving their service. For well-performing health plans it was probably difficult to improve their performance over the years given their high point of departure on several quality aspects. For instance, scores on reimbursement of claims can vary between 1 and 4. Above-average health plans had an average score of 3.8 in 2005, which is very close to 4, leaving little room for improvement.

An important question is what stimulates health plans to improve their performance. Three different mechanisms have been proposed to explain why public reporting of quality information would stimulate healthcare providers to initiate quality improvement projects [3,8,21]. First, identifying shortcomings may be sufficient to motivate professionals to improve their performance given their intrinsic motivation to provide service of high quality (*professionalism*). Second, comparable to the assumed effects of managed competition, the possible loss of market share can stimulate efforts to improve quality. Organizations then have an economical interest to excel in public reports (*market forces*). Last, it is held that healthcare providers value a good reputation and therefore do not want to be associated with bad-performing organizations in public reports *(reputation protection)*. The present results can not answer this question conclusively, but the finding that the overall performance of health plans did not improve more often for general points of improvement (as mentioned in the press-release) negates the idea that identifying shortcomings is sufficient to motivate health plans to improve the service they provide. Relatively bad-performing health plans did show more improvement than relatively good-performing health plans suggesting that health plans do not want to perform inferior compared to other health plans. In other words, as in previous studies [3,7,8,18], reputation protection appears to be an factor stimulating health plans to initiate improvement projects but fear for loosing market share is probably also an important issue.

In addition, it remains to be seen whether consumers or organisations interesting in a collective arrangement (for instance, employers or patient organisations) use the information on Internet to choose between health plans. One way to answer this question is to establish whether health plans that perform below average indeed loose market share. Unfortunately, these data were not available. In general, about 3-4% of the Dutch population switches health insurer each year. This percentage is comparable to the switching rates in other countries such as Germany (4-5%) and Switzerland (5%) [10,22,23]. Studies have also revealed that consumers use quality information when choosing a health plan and that they tend to choose better performing health plans [3,9]. It is, however, unknown whether a switching rate of 3-4% is enough for managed competition in health care to succeed [24].

Some limitations of the present study have to be noted. For one, a response bias occurred in all the four questionnaire studies on consumer experiences. Elderly and women responded more often than younger people and men. Previous studies have revealed that older people report more positive experiences than younger people; no consistent differences have been reported for men and women [25,26]. This means that the average performance of the health plans is probably overestimated in the questionnaire studies. Fortunately, the response bias was present in all the four years the consumer experiences were measured. This limitation thus probably did not affect our conclusions concerning the changes in performance over the years.

It is also important to note that the changes we found over the years are small. Although some differences are statistically significant, we have to ask our selves whether we can derive policy implications from these changes. In addition, the design of the present study did not allow us to determine whether the introduction of managed competition and the publication of comparative quality information were responsible for the observed changes in performance.

Future research should examine whether and for what reasons health plans initiate improvement projects. Ideally, experiments should be carried out in which health plans are randomly assigned to one of several conditions: only a confidential report, only a public report or a combination of a confidential and public report on their performance. At the same time, it has to be determined how long it takes for improvement efforts of health plans to be implemented and to translate into more positive experiences of consumers. In addition, studies that investigate whether consumers use the comparative quality information when choosing a health plan and whether relatively bad-performing health plans indeed loose market share are essential.

## Conclusion

The results concerning the effects of managed competition and the publication of comparative quality information on the performance of Dutch health plans are mixed. The overall performance of health plans did not improve over the years for all quality aspects and the improvements were also not more profound for quality aspects that needed improvement most. Health plans whose performance was below average in the first year (2005), however, did improve their performance over the years more often than health plans with an average or superior performance in that year. To determine whether managed competition in the healthcare system leads to quality improvement in health plans, researchers should examine whether and for what reasons health plans start improvement efforts.

## Competing interests

The authors declare that they have no competing interests.

## Authors' contributions

MH participated in the questionnaire studies, drafted the manuscript and contributed to all other aspects of the study. PS performed the statistical analyses. JR and DD contributed to the acquisition of the data, drafting the manuscript and critical revision of this manuscript. All authors read and approved the final manuscript.

## Pre-publication history

The pre-publication history for this paper can be accessed here:


